# The development of the concept of return-on-investment from large-scale quality improvement programmes in healthcare: an integrative systematic literature review

**DOI:** 10.1186/s12913-022-08832-3

**Published:** 2022-12-07

**Authors:** S.’thembile Thusini, Maria Milenova, Noushig Nahabedian, Barbara Grey, Tayana Soukup, Kia-Chong Chua, Claire Henderson

**Affiliations:** 1 King’s College London, London, UK; 2 South London and Maudsley NHS Foundation Trust, London, UK

**Keywords:** ROI, Quality Improvement, Value, Benefit, Mental health

## Abstract

**Background:**

Return on Investment (ROI) is increasingly being used to evaluate financial benefits from healthcare Quality Improvement (QI). ROI is traditionally used to evaluate investment performance in the commercial field. Little is known about ROI in healthcare. The aim of this systematic review was to analyse and develop ROI as a concept and develop a ROI conceptual framework for large-scale healthcare QI programmes.

**Methods:**

We searched Medline, Embase, Global health, PsycInfo, EconLit, NHS EED, Web of Science, Google Scholar using ROI or returns-on-investment concepts (e.g., cost–benefit, cost-effectiveness, value). We combined this terms with healthcare and QI. Included articles discussed at least three organisational QI benefits, including financial or patient benefits. We synthesised the different ways in which ROI or return-on-investment concepts were used and discussed by the QI literature; first the economically focused, then the non-economically focused QI literature. We then integrated these literatures to summarise their combined views.

**Results:**

We retrieved 10 428 articles. One hundred and two (102) articles were selected for full text screening. Of these 34 were excluded and 68 included. The included articles were QI economic, effectiveness, process, and impact evaluations as well as reports and conceptual literature. Fifteen of 68 articles were directly focused on QI programme economic outcomes. Of these, only four focused on ROI. ROI related concepts in this group included cost-effectiveness, cost–benefit, ROI, cost-saving, cost-reduction, and cost-avoidance. The remaining articles mainly mentioned efficiency, productivity, value, or benefits. Financial outcomes were not the main goal of QI programmes. We found that the ROI concept in healthcare QI aligned with the concepts of value and benefit, both monetary and non-monetary.

**Conclusion:**

Our analysis of the reviewed literature indicates that ROI in QI is conceptualised as value or benefit as demonstrated through a combination of significant outcomes for one or more stakeholders in healthcare organisations. As such, organisations at different developmental stages can deduce benefits that are relevant and legitimate as per their contextual needs.

**Trial registration:**

Review registration: PROSPERO; CRD42021236948.

**Supplementary Information:**

The online version contains supplementary material available at 10.1186/s12913-022-08832-3.

## Introduction

Quality Improvement (QI) programmes are being increasingly used to improve care quality in healthcare organisations [1]. QI is defined as a methodical approach to making improvements in a number of healthcare service aspects [1–3]. In particular, large-scale QI programmes are used to effect organisational level outcomes e.g., financial, and patient outcomes or even health system level outcomes e.g., population health [4, 5]. Large-scale QI combines various strategic elements into a coherent improvement process to improve safety, quality, capability, and capacity of organisations [4, 5]. Some QI programmes are delivered through local, national, or international collaboratives. A QI collaborative (QIC) combines multidisciplinary teams from different organisations to test solutions, and share learning in a specific clinical or operational area [6]. Whatever the type, QI programmes can have significant cost implications [7].

Investing in a QI programme may redirect money from other healthcare initiatives. In economic terms, this is called an “opportunity cost”, roughly meaning a lost opportunity for alternative investment [8]. For this reason, healthcare leaders must justify investments made or proposed for particular programmes. This is called making a QI business case. Return on Investment (ROI) is one such justification tool, used to decide how best to allocate limited healthcare funds [9, 10]. Investment allocation decisions have ethical, moral, political, and equity implications [11, 12]. Thus, the need to understand the meaning of ROI in the context of healthcare QI.

ROI is a financial tool that forecasts financial returns or profit from an investment [13, 14]. The forecasting process is called ROI analysis. This uses a methodology to convert (monetise) costs and benefits into ROI [15–17]. ROI is reported as metric (percentage or a ratio), e.g., ROI = 1:1 means a 100% return was made. ROI is one of many financial metrics used to judge efficiency of an investment [32]. ROI can be viewed independently, in comparison to other programmes, or against the counterfactual (doing nothing) [18]. In healthcare, ROI has been used to evaluate financial value of a programme post implementation [16]. ROI has also been used commercially as an economic performance measure for meeting product quality specifications [19]. ROI is sometimes used as a performance management tool, that is to ensure that organisations achieve their desired strategic goals [20]. These traditional definitions and uses of ROI are not disputed here. However, as ROI moved from commerce to healthcare frontlines, it became more than a metric. It became a concept of returns or gains from an investment.

ROI’s introduction into healthcare has caused concern [11, 21]. As well as the rationale for ROI being to justify investment business cases [22], the language also used is to “defend” against disinvestment [11, 23]. In many industries, including healthcare, there are several ROI technical and philosophical challenges [21, 24–28]. The major concerns are ROI’s de-emphasizing of wider organisational benefits. Modifications of ROI methodology have been attempted, for example detailing non-monetisable programme benefits as *additional* (not primary) benefits [9, 16]. This was in recognition that only a small fraction of QI benefits are actually monetisable [9]. However, there is still a general belief that only monetisable benefits should be seen as ROI [9, 16, 29]. This is in-spite of the recognition that non-monetisable benefits are highly valued by most organisations [9, 16]. This has created scepticism as to the extent of  the influence of non-monetisable benefits on investment decision-making [11, 30, 31].

Unsurprisingly, some deem the current ROI approach as aesthetic and synthetic [23], an insincere “placebo” and an oversimplification [27, 32]. As such, some industries appear to have rebranded ROI. In marketing and commercial service industries there is return-on-quality [33], and value-on-investment [34]. In healthcare, Leggat [35], called for a return-to-care, and Fischer & Duncan [36], a return-to-value. Healthcare researchers have also been slow to embrace ROI. Currently, many published QI-ROI studies involve small projects, often in health promotion, public health, or back-office services like laboratories (e.g., [21, 37–40]. This calls for an understanding and reconciliation of healthcare views on ROI. This endeavour must be based on a logical assessment of ROI as a concept, not as a metric. Further, this must be driven by a logic of contextual appropriateness [41].

Studying concepts such as ROI in context invites the understanding of institutional logics. Logics are socially constructed sets of assumptions, values, and beliefs that are used to ascribe meaning, as well as frame reasoning and legitimise choices [42]. As such, they reflect embedded cultures. Healthcare is a complex social environment, filled with sub-cultures [43]. Thus here, complexity constitutes multiple actions and interactions of not only humans, but technologies, processes, and systems [44, 45]. Healthcare has various stakeholders or groups and individuals that affect and are affected by healthcare [46]. These stakeholders have multiple, at times conflicting objectives and values [47]. Therefore, contextual interactions also entail multiple embedded theories, cognitive or symbolic systems [41, 47, 48]. For this reason, QI in healthcare is a complex intervention, with varied emergent and unpredictable outcomes [49].

Healthcare is at the juncture of many logics, primarily scientific, clinical, social, and economic logics [41, 47, 48]. Medical professionals may use science logic by emphasising a curing by focusing on evidence-based medicine, and or a care logic by focusing on interpersonal aspects. Managers may use an economic logic and focus on competition, markets, and financial outcomes. Further, some may use societal logics and focus on population health and socio-economic outcomes [41, 47, 48]. The presence of multiple logics explains the multiple ways used to define healthcare quality [48]. For example, some may describe quality as that which save costs; economic logic [50], is evidence based; science logic, or prioritises positive patient experience; care logic [51].

In the current study, the interest is the conceptualisation of ROI as a concept that is meaningful for healthcare stakeholders, particularly healthcare leaders as decision-makers. Concepts are mental abstractions which package complex meaning [52], and must be unpacked (or analysed) for effective application [53]. In this endeavour, concept analysis must be part of development of testable and practical theories [54]. Through concept analysis, scholars can produce evidence of their best estimate of the ‘probable truth’ about concepts [55]. This is a complex entangled task of concept analysis and development. In modern philosophy such as Critical Realism [56], this undertaking assumes that concepts are contextual and changeable [54, 55]. Further, this presupposes moderate philosophies about the nature (Ontology) of concepts [54].

Moderate philosophies are different from traditional philosophies where the ‘truth’ is seen as absolute and or residing on one end of the spectrum. In Realism, reality exists regardless of human perception, whilst Relativism views reality as based on human perception and socially constructed. Lack of clarity about concepts can lead to poor communication, poor application in research and in practice [52, 57]. As healthcare organisations are complex dynamic contexts, modern philosophy insights could support relations between QI implementers and investors. A scientific study of ROI as a concept grounded on a moderate philosophy may help engage QI researchers, improve practical application for practitioners, and improve communication amongst improvement stakeholders.

## Aims

The aim of this study was to learn how the concept of a return-on-investment for healthcare large-scale QI is understood, and how this differs from related concepts. We first analysed, then developed the large-scale QI-ROI concept for healthcare based on the systematic literature review. We then proposed a framework for analysis of return-on-investment from QI programmes.

## Methods

This paper is part of a larger integrative systematic literature review on the conceptualisation of ROI in healthcare QI. Our review is registered with PROSPERO, CRD42021236948. A link to our PRISMA reporting checklist [58] can be found in the supplementary files. We followed review guidance by Whittemore and Knafl [59] and conceptual analysis and development by Hupcey and Penrod [55] and Jabareen [53]. This led to 8 separate review stages. Stage 1; clarifying research question, involved background reading as discussed in our protocol on PROSPERO. The remainder of the stages are reported here. Stages 2–3 involved searching and selecting literature. In stage 4 we assessed the quality of research studies, stages 5–8 are reported in the synthesis, analysis, and results sections below.

### Search strategy

The identification of suitable search terms was an iterative processes. To compile a list of ROI-like terms, we referred to the National Institute of Health and Care Excellence (NICE) ROI guide. NICE [60] views ROI as a term for various economic evaluation tools and processes used to evaluate value-for-money of healthcare programmes. Economic evaluation is the comparative analysis of alternative courses of action in relation to both their costs and consequences [61]. Economic evaluation methods include cost–benefit analysis (CBA), cost-effectiveness analysis (CEA), and cost consequence analysis (CCA). Search terms were also derived from background reading in healthcare and commercial literature. The final search terms were in three categories (Table 1): (i) context, (ii) QI methods, and (iii) QI outcomes. Category 2 terms were the most frequently mentioned QI methods in literature. Category 3 terms denote some form of outcome (return, benefit) derived from some form of input (investment, cost, resource).

**Table 1:** Search terms

CONTEXT	AND	QI METHODS	AND	QI OUTCOMES
Health		Quality improvement OR QI OR statistical process control OR Lean OR Six sigma OR Lean Six-sigma OR Audit and feedback OR Model for improvement OR Root cause analysis OR Process mapping OR Define Measure Analyse Improve Control OR DMAIC OR Plan do study act OR PDSA OR PDCA OR Driver diagram OR Theory of change OR Logic model OR SPC OR statistical quality control OR SQC		Return on investment OR Rate of return OR Payback OR Business case OR Benefit cost OR Risk benefit OR Cost benefit OR Cost consequence OR Cost reduction OR Cost containment OR Cost control OR Cost avoidance OR Cost saving OR cost outcome OR Value on investment OR Value care OR Value for money OR Value improvement OR Improvement outcome OR Resource outcome OR Resource benefit

We searched Medline, Embase, Global health, PsycInfo, EconLit, NHS EED, Web of Science, Google, Google scholar, organisational journals, as well hand-searched citations. No language and date limits were set to enable us to note any changes in QI-ROI conceptualisation over time. The search ended January 30, 2021. An example of the search strategy for Web of Science has been provided as Supplementary Table 1. A link to more of our search strategies can be found in the Supplementary files. The main search terms are defined in Table 2.

**Table 2:** Definitions of terms

Terms	Description
CEA	Cost-effectiveness analysis: Achieving more of the outcome for the same cost or achieving the same outcome for less cost, expressed in incremental benefits on Quality Adjusted Life Years (QALY), or incremental cost-effectiveness ratio (ICER)
CUA	Cost-utility analysis: Similar to CEA but for multiple outcome measures in quality-of-life units (QoL)
CBA	Cost–benefit analysis: Financial expression of costs and benefits from a programme in a cost–benefit ratio (CBR) CBA is the basis for ROI and SROI; CBA and SROI are societal perspectives, ROI is managerial/investor focused
ROI	Return on Investment: Expression of costs and benefits from a programme expressed in an ROI metric
SROI	Social Return on Investment: Expression of costs and benefits from a programme expressed in a ROI metric Includes benefits for society, environment, and others. Engages various stakeholders in the calculation process
CCA	Cost consequence analysis: comparing alternative interventions or programs in which the components of incremental costs and consequences without aggregating these results Economic terms sources: [17, 60–63]
Value Value for money	Any outcome seen to be of importance, utility, or usefulness. [64] Obtaining the most useful (utility), most effective, and less wasteful (efficient) from your service or purchase [60]
Benefit	Any outcome that produces useful, helpful, or advantageous outcomes [65]
Outcome	A result or consequence of an action or process [66]
QI methods	Methods used to improve organisational processes and behaviours e.g., PDSA, Lean, Six-Sigma, Lean-Six Sigma, Audit & Feedback. [67–69]
Healthcare organisation (UK)	A unique framework of authority within which a person or persons act or are designated to act towards some purpose as a direct provider of healthcare services (preventative, curative, rehabilitative, or palliative). Includes Local Authorities with Social care working in cooperation with the NHS [70]

### Eligibility

**Fig. 1:**
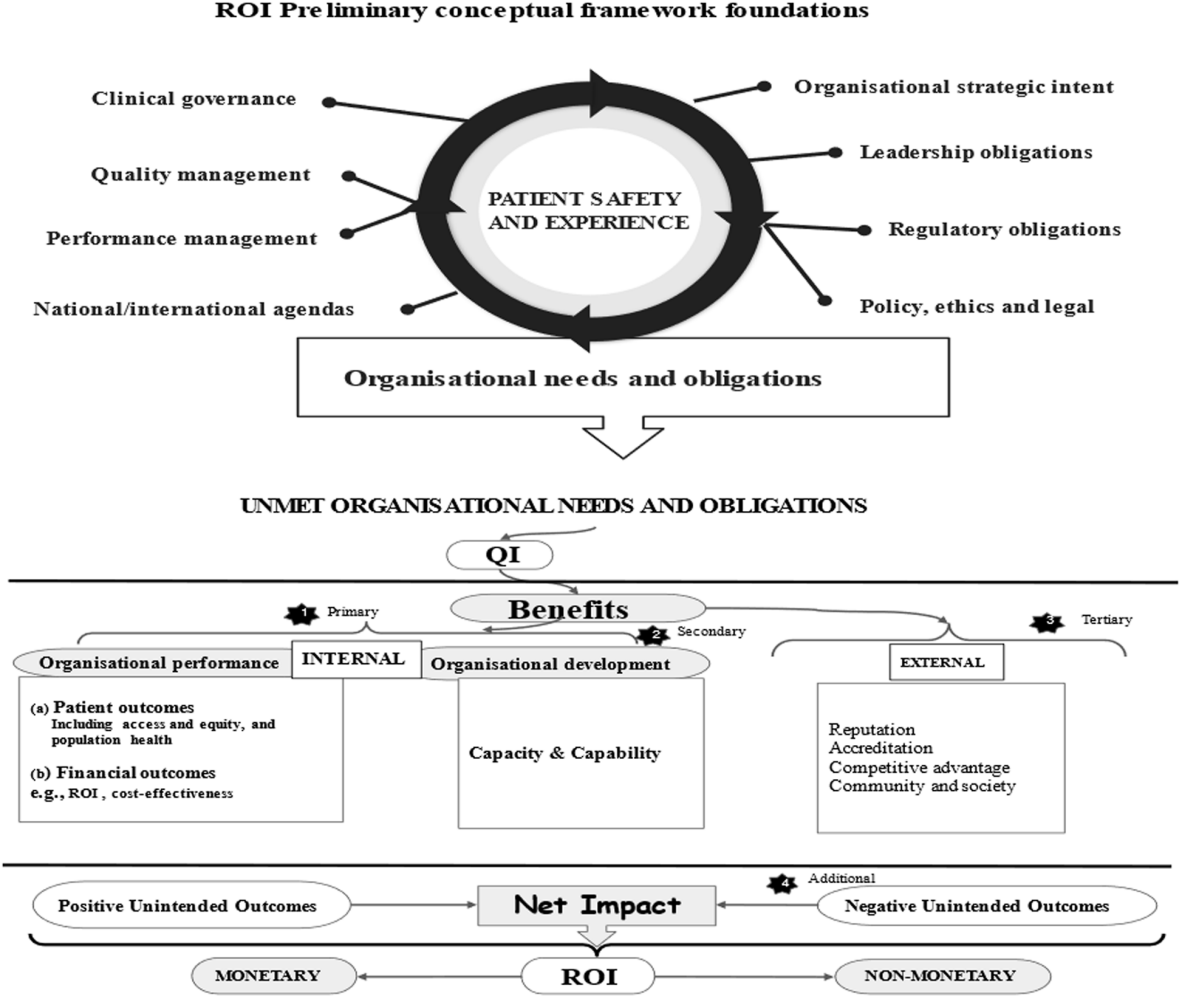
Preliminary QI-ROI Conceptual Framework

As ROI is an investment allocation decision tool, our stakeholder of interest were the healthcare leaders, and level of analysis the organisation, where decision-making outcomes are assessed. During our initial search, many articles identified themselves as large-scale QI programmes. However, at closer inspection, some of these only impacted a small part of an organisation and were therefore equivalent to a small organisational unit intervention. To focus our selection criteria, we developed a preliminary ROI conceptual framework ( Fig. 1). The preliminary framework contained various needs and obligations of healthcare organisations [71, 72]. We assumed these to signal desired organisational outcomes.

The Framework had four criteria: 1) organisational performance (patients and financial outcomes), 2) organisational capacity and capability, 3) external relations (e.g., accreditation), and 4) unintended consequences (positive/negative). Organisational performance is a marker of how well organisations perform on delivering value for its stakeholders [73]. Thus, in a way it includes external relations, e.g., population health. However, external outcomes were isolated here to deduce some unique external outcomes and obligations towards external stakeholders. We included negative outcomes as potential indicators of the lack of ROI positive returns. We then used this framework to decide on eligibility.

To be included, the literature that had to mention at least three QI organisational goals or benefits, two of which had to be patient or financial outcomes. Through this, we sought to isolate articles that discussed a range of QI outcomes, with patient and financial outcomes as basic organisational goals. In addition, articles had to mention use of at least one QI method, and involvement of various stakeholders, in at least two organisational units. Altogether, this denoted a three-dimensional criteria: depth, breath, and complexity of QI programmes per organisation. We included literature on discussions of large-scale QI programmes outcomes across healthcare globally. Table 3 has included/excluded article types.

**Table 3:** Eligibility criteria and selected article types

Eligibility	**Outcomes** QI Effectiveness or process outcomes e.g., goals achieved QI economic outcomes e.g., savings Clinical outcomes e.g., symptoms Organisational outcomes e.g., development Short-term, intermediate, long-term, and impacts	**ROI concepts** Cost-effectiveness Cost–benefit Value Benefits QI outcomes/consequences	**Level of analysis** Organisation **Type of literature** Empirical and non-empirical reports Conceptual and Grey literature
Included	**Large scale complexity, depth, and breadth** At least one QI method used At least three organisational outcomes At least two organisational departments engaged		
Excluded	Articles where one department was engaged, two or less organisational outcomes were reported, and pre-prints		

### Screening and selection of articles

Data were managed using Endnote citation manager [74] and Ryann systematic review app [75]. Screening and selection were performed by two independent reviewers, ST, and MM. To refine our selection criteria, five articles were initially selected and discussed to clarify any uncertainties. The two reviewers then completed the screening and selection of the remaining articles independently: ST 100%, MM 5%. Overall agreement was over 90%. Disagreements were discussed and settled by ST and MM, as well as with co-authors CH and K-CC.

### Quality assessment

For researchers of integrative reviews and conceptual development, quality assessment is optional as the quality of studies has little or no bearing on concept development [53, 59]. As such, there was no intention to exclude articles based on their quality. However, to understand the scientific context in which QI benefits are discussed, we assessed all empirical studies using specific quality assessment and reporting tools. For reviews, we used the  Critical Appraisal Skills Programme (CASP) tool [76], for mixed methods, we used the Mixed Methods Appraisal Tool (MMAT) [77], for implementation studies, we used Standards for Reporting Implementation Studies (STaRI) [78]. For economic evaluations, we used the Consolidated Health Economic Evaluation Reporting Standards (CHEERS) [79], and for QI, we used the Standards for QUality Improvement Reporting Excellence (SQUIRE) tool [80]. As these are different tools, there was no single criteria to judge collective study quality. We therefore assessed the number of appropriate items reported or addressed as per respective study’s tool. We assigned good if 80–100% items were addressed, moderate if 50–79% of items were addressed, and poor if less than 50%.

### Data extraction

Data extraction was performed using words and phrases in the preliminary conceptual framework as well as outcomes in the reviews search terms. We searched for these from all parts of an article where QI benefits, outcomes, and goals may be discussed. Articles were tabulated according to type of article, type of focus, country, setting, programme type, and outcomes discussed. The data collection tool can be found as Supplementary Table 2.

### Data synthesis and analysis

The synthesis and analysis section forms stages 5–7 of the integrative review process (integrate, synthesise, analyse). The synthesis, analysis, and framework development were performed iteratively by ST. All steps in the synthesis and analysis were discussed with co-authors CH and K-CC. We used the principle-based analysis method [55], to assess the maturity of the QI-ROI concept in healthcare literature. This involved asking four principle-based questions: 1) Epistemological principle: is the concept clearly defined and well differentiated from other concepts? 2) Pragmatic principle: is the concept applicable and useful within the scientific realm of inquiry? Has it been operationalised? 3) Linguistic principle: is the concept used consistently and appropriately within context? 4) Logical principle: does the concept hold its boundaries through theoretical integration with other concepts?

Once the ROI concept maturity was established, we followed Jabareen’s [53] conceptual framework development process. Jabareen describes a conceptual framework as “a network…of interlinked concepts that…provide a comprehensive understanding of a phenomenon or phenomena” (p.51). A framework was developed by identifying and naming concepts, describing concepts. Concepts were then categorised according to their ontological, epistemological, or methodological role. This was followed by synthesising, sense-making, and integration of similar concepts into one new concept, the QI-ROI. We also contextualise the ROI concept by highlighting how the concept is defined in the healthcare context, the alternative explanations afforded by the new concept which are not normally enabled by similar concepts, and the patterns in which the QI-ROI concept appear in the healthcare context [54].

## Results

**Fig. 2:**
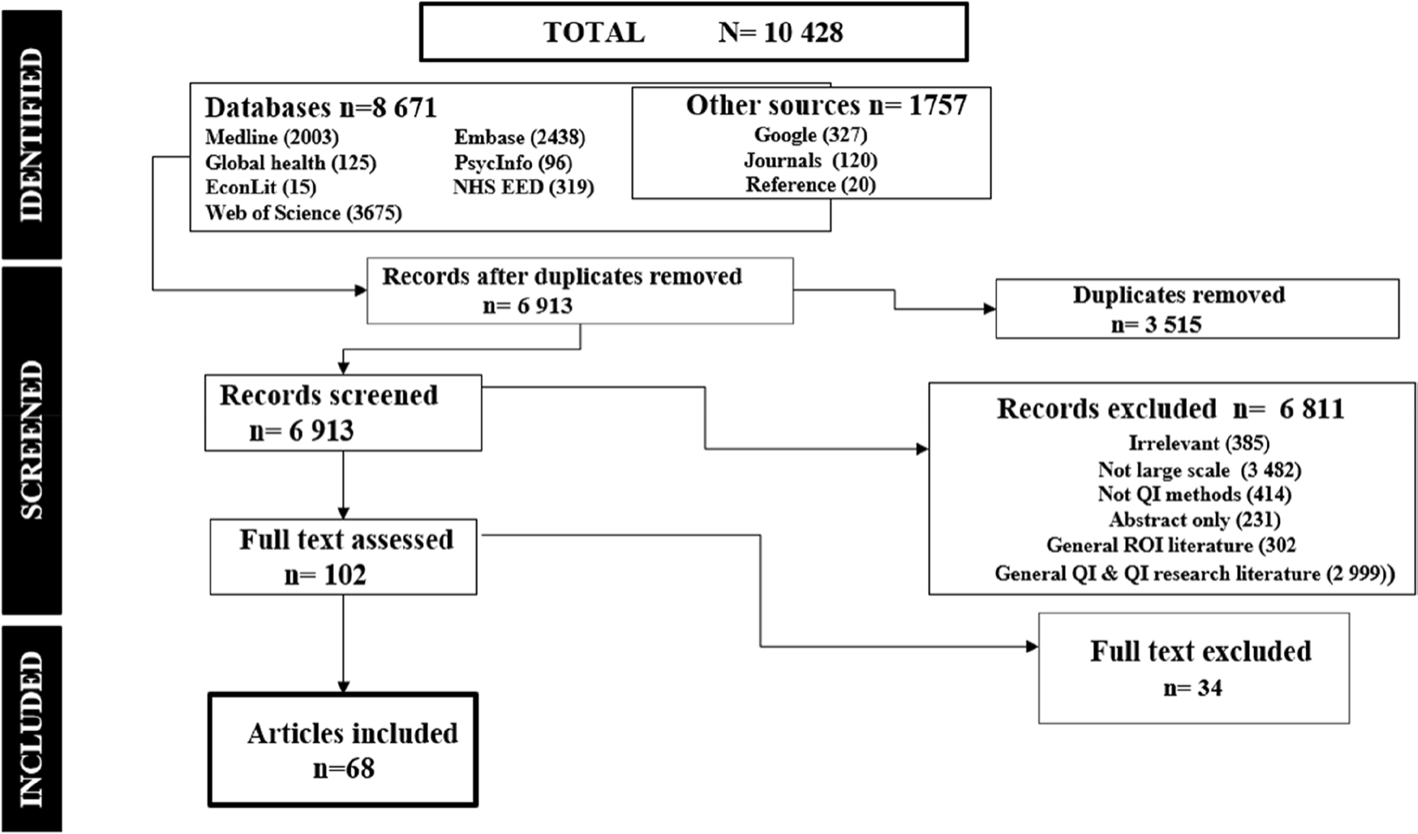
PRISMA Flow-chart

A total of 10 428 articles were retrieved, 10 327 were excluded for various reason as seen in Fig. 2. One hundred and two (102) articles were eligible, 34 were excluded and 68 included. Included articles were: Conceptual *n* = 24, Quantitative *n* = 19, including three economic evaluations (CEA *n* = 1, Economic Impact *n* = 1, ROI *n *= 1)**,** Qualitative *n* = 3, Mixed-Methods *n* = 8, Systematic Reviews *n* = 8 (2 economic; 1 SROI), Literature reviews *n* = 2, Brief Report *n* = 4, Thirty three of the excluded articles engaged a single department and/or discussed two or less QI outcomes/goals. Thirteen of these were collaboratives. There was one pre-print. A link to the excluded studies document is available on the supplementary files.

### Article characteristics

Included articles covered different healthcare levels and disciplines globally. Primary care included public health, child and maternal health, and mental health. Secondary or tertiary healthcare included mental health, medical and surgical care, critical care, accident and emergency and acute care services, paediatrics and neonatal care, outpatients, pharmacy, and laboratories. One article covered both health and social care, and another was about a charitable organisation. Global regions were Africa, Asia, Europe, Australia, and Canada, with the US and UK the mostly represented. The summary of included studies can be found in supplementary files (Supplementary Table 3).

### Quality of studies

From the 68 articles, 30 were not subject to quality assessment. This included conceptual articles, unsystematic literature reviews, brief reports. Thirty eight articles were subject to quality assessment: 19 quantitative studies, three qualitative studies, eight mixed-methods studies, and eight systematic reviews. Of the 38, 39% reported or addressed 80%-100% all items required, 43% reported on 50%-79% the data required, and 18% reported below 50% of items by their respective reporting tool. Thirty out of thirty eight studies were primary studies. In these, there were three main areas of poor reporting and or poor rigour on all types of studies: ethics (29%), statistical analysis methods (75%), discussion of study limitations and their management (42%), integration of quantitative and qualitative data unclear (29%). Reporting of funding and affiliations missing on three of all 35 studies. Therefore, the quality of the studies was summed up as moderate. The quality assessment summary is available as Supplementary Table 4.

### Synthesis summary

#### QI Economically-focused literature

Economically-focused literature were those articles whose specific focus was on either studying or discussing QI economic benefits. This made up 15 articles of the 68 QI literature. Amongst these were four conceptual literature, (three of which were business case discussions) [22, 36, 81, 82], four QI evaluation frameworks [83–86], two systematic reviews of economic evaluations [87, 88], one article discussed cost effectiveness of QI programmes [89], one article calculated an organisation’s QI related cost-savings [90], and two were economic evaluations [91, 92]. One article discussed cost–benefit analysis [93]. Of the total 15 articles, ROI was a specific subject of only four [81, 84, 87, 91].

1. Business case conceptual literature
  Conceptual literature authors discussed QI business case development [22, 36, 81, 82]. Their views were based on literature reviews, expert opinions, case studies, or all three. This literature were mostly sources of information for how to develop business cases that justify QI programme from a financial benefit perspective. However, they also highlighted multiple QI objectives and stakeholders. As such, there was a requirement to present QI outcomes as a monetised ratio, and or use of ROI as a financial performance measurement method [81]. However, this literature also highlighted multiple QI objectives as well as multiple stakeholders as QI beneficiaries.
2. ROI Frameworks
  These authors advanced on the conceptual literature by developing QI business case frameworks that incorporate monetary and non-monetary benefits [83–86, 93]. QI was seen to serve various organisational interests, for various internal and external stakeholders. As such, business case frameworks centred around these principles. Swensen et al. [86]focused on four organisational interests: patient’s needs, organisational reputation, pride, and financial returns. There was also an interest in organisational productivity and efficiency. Bailit and Dyer [83], described 10 business case arguments that combine financial, strategic, and internal organisational objectives.
  Shah and course [85] had a six category framework containing three as financial measures (revenue, cost-reduction cost-avoidance) and one measure for patients, family and carers experience, and one for staff experience, and lastly one for productivity and efficiency. Financial objectives and outcomes included cost-avoidance such future legal costs [86]. Internal outcomes also included capacity building, whilst external outcomes examples included market share [84–86].
3. QI economic evaluation literature

There were three economic evaluations [89, 91, 92], and two systemic reviews of QI economic evaluations [87, 88]. By the virtue of their study foci, their measure of ROI was the monetary. These authors saw savings as an important QI outcome, however also discussed a wide range of QI benefits. For example, Crawley-stout et al. [91] considered internal outcomes (e.g., cost-reduction, productivity, and time savings) and external benefits (e.g., patient costs and carer time). Crawley-stout et al. described ROI as a performance measure used to evaluate investment efficiency in financial terms. de la Perrele et al. reported a lack of QI economic evaluations in their review. They concluded that collaboratives are potentially cost-saving. However, they found that studies used variable methods to assess cost and effectiveness, and that studies did not report negative findings. They recommended that future research should include societal perspectives of costs and savings [88]. Banke-Thomas et al. stated that SROI (a societal version of ROI) can be used across healthcare [87]. However, there were challenges with inadequate skills for ROI evaluation, lack of credible financial proxies, a lack of consensus on; who to include as beneficiaries, how to account for counterfactual and appropriate study-time horizon [87].

#### QI non-economically focused literature

These made up 53 [94–147] of the selected 68 articles. These articles included QI effectiveness, process, and impact evaluations as well as discussions of QI achievements over time e.g., [94, 110, 116, 117, 130, 138]. These articles did not focus on ROI or economic measures, but nonetheless highlighted financial outcomes as important benefits for consideration. Some QI implementation studies assessed their implementation costs [109, 140, 145], as part of their study reporting guidelines [78, 148]. Authors here also discussed improving QI effectiveness determinants such as staff and safety culture development. Authors discussed or mentioned financial value or benefit, financial returns or outcomes, cost savings, reduction, containment, and economic impact, as well as productivity, efficiency, value, and benefits. Of these, cost-saving was the most frequently used term. These articles considered ROI as one of many organisational outcomes [103, 130, 135].

There were three QI evaluation frameworks [103, 104, 124]. These frameworks also considered various elements of organisational benefits. Chow-Chua and Goh [103] combined existing organisational performance tools; the Singapore Quality Award (SQA) model (modelled after Baldrige Award) and balanced scorecard (BSC) to develop a performance and quality improvement evaluation framework for hospitals. Four strategic components were seen as crucial: the drivers of QI (e.g., leadership), internal performance, knowledge management, and QI outcomes. McLees et al.’s [124] framework for QI in public health was described as a performance management tool, and focused on two key constructs: efficiency and effectiveness. Ciarniene et al.’s [104] framework envisioned broad value creation through QI.

#### Integrated synthesis

Morganti et al. [126] remarked that there is a lack of an agreed concept of QI success, and by extension, ROI. This was seen in how authors gave priority to certain outcomes. Van den Heuwel et al. [141] for an example, referred to quality improvement as business improvement, viewed ROI quantitatively, and expected quality improvement to be a valuable “side effect” of value improvement (often a euphemism financial improvement). Alternatively, others proclaimed to value patient safety and quality first, and saw financial matters as *the* valuable “side-effect” of QI [85]. Hunter et al. [116] considered “cost savings or increased efficiency “helpful by-products” (p. 129).

Swensen et al. [86] QI business case discussion stated that their QI investment decisions were never based purely on positive ROIs but on broader qualitative considerations. A similar view was held by O’Sullivan et al. [130] and Shah and Course (2020). Bailit and Dyer [83], advocated for broad business cases that embrace different rationales for QI investment. Fischer and Duncan [36] stated that some interventions are purely designed to produce health outcomes. They also called for a broader QI outcome definition that acknowledges the utility of differing projects and value for all stakeholders. The review also indicated that even failed goal attainment can be useful in providing insights and legacies like building capacities and safety cultures [94, 110, 116, 117, 130, 138, 146].

Overall, financial outcomes were not the primary or commonly sought goal or addressed outcome. However, it was seen as directly or indirectly significant by the majority of the authors. The perception that QI is an expense used for revenue generation was seen to be due to faulty assumptions by some authors [86]. For example, authors suggested that profit-seeking through QI first emerged as an optional strategy to increase revenue and market-shares by for-profit healthcare organisations [22, 83, 86]. However, grey areas on views existed and views appear to have shifted towards integrating or emphasising non-monetary outcomes over time. The literature also agreed that QI does not always save cost, and financial outcomes are not the only organisational objectives [22, 36, 82, 83, 89, 93]. These literatures portrayed ROI as any value or benefit from QI for various stakeholders.

**Fig. 3:**
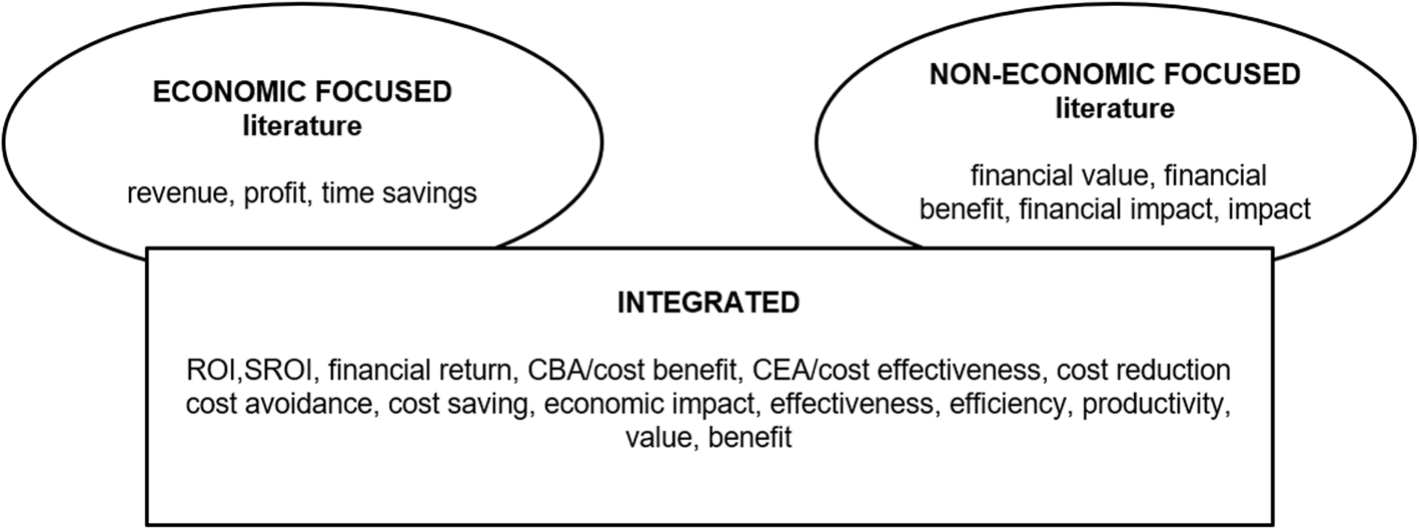
ROI-like concepts

The reviewed literature illustrated five main ROI uses related to QI: ROI as 1) a strategic business case development tool, 2) an investment performance measure, 3) a comparative evaluation tool, 4) a cost management tool, and 5) a performance management tool. ROI was also used to create fiscal awareness [81]. Some of this ROI use was similar to commercial ROI use. Various concepts were used to denote a return-on-investment. These concepts were used in relation to changes and improvements in various organisational outcomes including patients, staff, financial, and overall organisational development. Both economic and non-economic focused literature used almost identical concepts to denote an investment and a return as seen in Fig. 3.

However, profit, revenue, and market share were mainly found in the few economic focused literature. The majority of literature put greater emphasis on non-monetary QI benefits. This indicates that although different logics were applied in the conceptualisation of healthcare QI-ROI, the dominant logic was that of health and social care and not economics or markets.

At face value, there were two broad QI-ROI philosophies: the economic and the healthcare ROI philosophies. However, our synthesis indicated a merging meaning of ROI from different schools of thought. Through markets logic, the economic philosophy perspective views outcomes in terms of their tangible, quantitative, or financial offerings. The economic philosophy is related to managerial logics as managers’ roles require them to monitor organisational financial performance. Alternatively, the healthcare perspective suggested a more dulled though increasing financial focus. Healthcare logics (medical, societal) tended to view ROI qualitatively, primarily from a patient and staff perspectives but also wider internal and external stakeholders. The integrated literature indicated that healthcare leaders value these perspectives too. Table 4 illustrates this finding.

**Table 4:** ROI concept perspectives

Type of QI outcome: ROI-like concepts	Stakeholder perspective	Dominant logic	Philosophical perspective
**Organisational outcomes:** Improvement, efficiency, productivity, effectiveness, profit, financial return, ROI, SROI, CBA, CEA, economic impact, cost saving, cost avoidance, cost reduction, market share, reputation, organisational development, performance (organisational), management (cost)	Managerial	Markets/ Economic	Realism
**Patients, family, carer, and societal outcomes:** improvements, value, benefits, impacts (SROI, CBA)	Managerial Patients Society	Medical Societal	Relativism/Interpretivism
**Staff outcomes:** improvements, value, benefits, impacts	Managerial Staff	Medical Societal	Relativism/Interpretivism

## Discussion

Although concept analysis, concept development, and conceptual framework development are traditionally separate steps [55], they have been performed concurrently in this review. Firstly, we sought to understand the nature of the ROI concept in healthcare QI. Using the Hupcey and Penrod [55] principle-based concept analysis method, we found that the QI-ROI concept is not clearly defined or developed (principle 1), the ROI application method is unclear (principle 2), its appropriate use is unestablished (principle 3), and conceptual boundaries are unclear (principle 4). These determinations answered the first part of our research question; the nature of the ROI concept in healthcare QI.

We then sought to answer the second part of our research question; the definition of the QI-ROI concept and its relationship with similar concepts. We did this by following the concept development and conceptual framework development guide described earlier by Hupcey and Penrod [55], and Jabareen [53]. Our review confirmed that various concepts and terms are used to denote returns or investment vs returns in healthcare QI. Some of these concepts (e.g., CEA, CBA, value) were identified in the background literature and used as the review’s search terms. These concepts, including the differences between costs, investments, and revenue have been discussed in more details in economic literature (e.g., [61, 149–151]. We refer interested readers to such text for nuanced descriptions.

1. QI-ROI concept development

To develop the QI-ROI concept, we first eliminated similar concepts by differentiating QI-ROI from other ROI-like concepts and terms. To start with, financial or economic returns are alternative ways of saying ROI [60]. Other terms used to a lesser extent in the review were economic impact, which denotes only the cost of illness [152], and cost management which is a process of managing and controlling costs of a programme to fit desired criteria, e.g., to reduce costs [153]. Cost-reduction is described as resulting from providing the same or better quality for a lower cost, through new ways of working that eliminate waste ([178], p.4). Cost avoidance is cost-reduction related to preventing future costs [178]. Very few articles mentioned profit or revenue. Publicly funded healthcare such as the UK NHS does not customarily engage in profit-making. Net profit is what remains after all expenses have been subtracted from net revenue (income generated minus costs). The remaining concepts: SROI, cost–benefit, cost-effectiveness, productivity, efficiency, value, and benefit were seen as the most mimicking ROI. These concepts have varying degrees of association with ROI depending on the ultimate objective sought through a QI programme.

- (1a) ROI vs other economic evaluation methods

In health economics, cost-effectiveness is assessed using CEA and whilst costs-benefits are assessed using CBA. CEA and CBA goals are to ensure that fixed allocated quantity of healthcare resources result in most health outcomes improvement (CEA) or maximum social advantage (CBA) [61]. CEA and CBA denote cost vs goal achievement. Although conceptually different, in practice, researchers do conflate the different types of evaluation [154, 155] or see them as interchangeable [60]. Comparative ROI mimics CEA, but ROI reports an aggregated cost–benefit metric similar to CBA’s cost–benefit ratio (CBR). Alternatively, CEA reports an incremental cost-effectiveness ratio (ICER) per health outcomes. Incremental benefits using ROI of new QI programmes have been compared by some researchers [156, 157]. However, given the multiple healthcare objectives, these metrics represent only a fraction of programme benefits or consequences [15]. This point is supported by the current literature review.

CBA is the basis of ROI and SROI. SROI and CBA are monetisation of broad programme benefits and costs such as societal costs and benefits. SROI extends CBA by including environmental and other stakeholder benefits [37]. Alternatively, ROI generally focuses on programme specific costs and benefits from a managerial perspective [9, 16]. As returns-on-investments evaluation methods, CEA, CBA, SROI, and traditional ROI are too narrowly focused as they all ultimately only emphasise monetary focus. According to Bridges [101], CBA, does not account for how care is produced, and thus excludes many crucial organisational outcomes. Bridges suggested that what is needed is a systematic value assessment approach.

- (1b) ROI vs input–output based measures

CEA, CBA, productivity, and efficiency are similar as their goals are using resources without waste. However, they are all a single focus outcomes. CEA/CBA are an input vs goal measures, efficiency and productivity are input vs output measures. Productivity and efficiency are ROI-like as they denote a return (output) of an investment (input) [158]. Inputs and outputs may be both monetary and non-monetary. Productivity is the quantity of outputs per investment/input. Efficiency is achieving those outputs with least or no waste (e.g., in time, money, effort). Therefore, unlike CBA and CEA, efficiency and productivity are related to exactly how care is produced. For example, increasing productivity by increasing patients seen (output) per clinician (input), whilst providing quality care without wasting resources (efficiency).

Efficiency is divided into allocative, productive, and technical efficiency [61]. Simply put, allocative efficiency refers to allocation of healthcare resources such that the most benefits are delivered [159]. Productive efficiency is increasing output per given resource/input/investment (e.g., seeing more patients by same staff member). If this is done such that more is obtained from the same resource, or less resource is required for the same output, it is technically efficient [151]. This description also fits CEA, with outputs being effectiveness. It also mimics the concept of value-for-money (VfM), used to describe the optimal balance between efficiency, economy (lowest cost), and effectiveness [60]. Efficiency and productivity are crucial in healthcare as profit-based ROI is deemed improbable [160]. Efficiency can translate to both monetary ROI (e.g., savings), and non-monetary benefits (e.g., improved staff work experience).

Productivity and efficiency are often used to measure performance of healthcare organisations [150, 158]. Productivity may enable allocative efficiency of funds or better time allocation for tasks by staff. Productivity can be an efficiency measure (input/output) [158]. It can also be a combined effectiveness and efficiency measure (goal/input/output), or of all that makes an organisation function better [150]. The latter is what the reviewed literature indicated QI-ROI to be. Effectiveness through attainment of goals alone is therefore also insufficient to describe QI-ROI. Goals may be achieved, but inefficiently. In a balanced productivity-efficiency-effectiveness relationship, all three contribute to the overall QI-ROI [161]. This may then mean avoiding, reducing, and containing costs, and thus saving costs.

- (1c) ROI vs cost saving

Cost-saving is also a more likely outcome than hard-cash profit in healthcare QI [16]. Cost-saving was a particularly prevalent term in the reviewed literature and ROI in healthcare has been called savings [21]. The current desire to save costs is thought to have driven the change in focus from  cost-effectiveness studies to ROI [162]. Cost-saving means saving money that would have otherwise been spent. Savings (time/money) often result from better efficiency and productivity. Similar terms such as cost-containment, cost-minimisation, cost-avoidance, cost-reduction are also not seen here as complete representations of QI-ROI. Here, these terms are seen as representing outputs, initial or intermediate outcomes that lead to savings. These terms (including cost-management) can also be processes or abilities that enable cost-saving or profit-making. Together, these terms refer to mechanisms (or processes that enable an outcome) [163] through which long-term financial ROI may be achieved. Alternatively, some may see these initial outcomes as benefits themselves.

(2) QI-ROI framework development

**Fig. 4:**
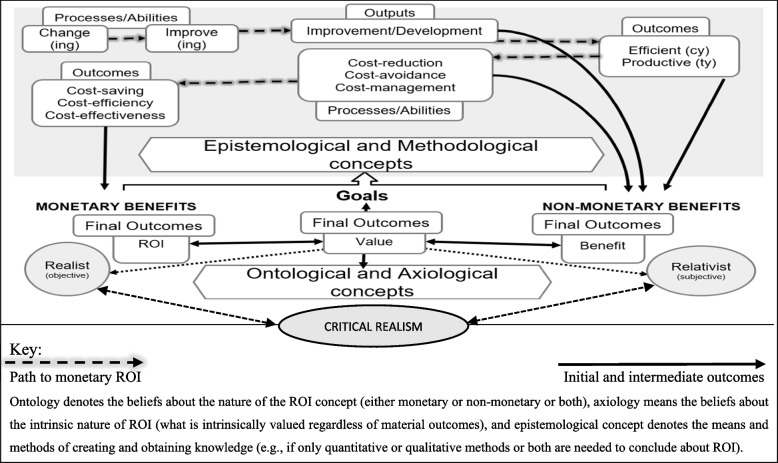
QI-ROI conceptual framework

For some organisations, initial outputs and intermediate outcomes may be the intended outcomes and therefore may represent a form of ROI. In Phillips et al. [16] for example, productivity and efficiency were viewed as final intended outcomes of improvements. In other instances, cost-effectiveness may be the intended goal. Often in healthcare the ultimate objective is to achieve higher goals, such as financial stability. In such cases, implementing QI leads to change and development and possibly improvement in desired outcomes. Improvement may result in improved productivity and efficiency. This in turn improves abilities to better avoid, reduce, or manage costs, leading to savings, and potentially monetary ROI. All of this is a benefit in and of itself. Monetary ROI depends on each output, ability, or outcome, most of which is non-monetary. This conceptualisation of QI-ROI is illustrated in Fig. 4 below.

The QI-ROI conceptualisation can be translated as follows: value is any outcome seen to be of importance, utility, or usefulness [64]; attaining a return-on-investment whatever that/those are, is valued and therefore of benefit. A benefit is any outcome that produces useful, helpful, or advantageous outcomes [164]. Any benefit is of value in of itself. Based on this review, a fuller description of QI-ROI is suggested as follows: QI-ROI is any value or benefit (or any valued benefit) derived from or contributed to by QI programmes. This value or benefit maybe in a form of an improved output, process, ability, outcome, and or overall impacts, depending on an organisation’s values and objectives.This description reflects the Healthcare Financial Management Association (HFMA), NHS England, and NHS Improvement (NHSE&I)'s view. The NHSE&I differentiates from cash-releasing benefits that enable resource allocation, and non-cash-releasing benefits that provide economic benefit, without releasing cash [178].

This definition is different from that which supports the economic logics. For example, Phillip et al. [16], and Solid [9] who discussed value *and* ROI as separate (presumed to mean non-monetary *and* monetary value). Here, only monetary value is seen as ROI. This assertion is based on viewing ROI as a purely quantitative metric. However, Solid’s writing also refers to value as being subjective (p. 5). The definition of ROI concept as any benefit may be deemed an abuse of ROI [16, 29]. However, this definition was based on the review of numerous healthcare stakeholders’ views. This may be an indication that new constructive conversations about how to integrate economic and accounting concepts in healthcare are needed [57, 165]. After-all, value was not always seen as a ratio between perceived benefits and perceived costs [166]. It was initially viewed as multi-dimensional by earlier economic scholars [57, 166–168]. Therefore, other QI programme effects that are regarded as valuable must not only be considered [61], but prioritised where appropriate.

The view of QI-ROI in healthcare as a broad and no-specific concept, encompassing both monetary and non-monetary outcomes, opens ROI to being a context-specific and dynamic concept. This is in-line with the moderate ontological expectations of modern concepts [54, 55]. This suggests abilities to compromise and accommodate varied logics that govern healthcare [169]. The concepts of ROI, value, and benefit, denote the beliefs about what is a true return, value, or benefit (Ontology) as well as what seen as a moral good (Axiology) in healthcare QI. As such, the QI-ROI can be seen as a value-based ROI. Traditional ROI is a monetary measure (Realism), benefits in general tend to be described as non-monetary (Relativism), and value can denote either a monetary or a non-monetary outcome (Critical Realism). These beliefs then influence how evidence is created, viewed, and studied (Epistemology and Methodology) [53].

The lack of convincing vocabulary to argue against the logics of the markets in healthcare was seen as the limiting factor by Bozeman [170]. In the current review this has also been demonstrated. In non-economic literature, financial outcomes mentions appeared to be nebulous, or in general use of terms as in everyday language. Authors here focused on non-monetary outcomes. They discussed aspirations to raise fiscal awareness and encourage financial outcomes focus on QI evaluations. This can be contrasted with economic focussed literature where for example, economic evaluations referred to ROI as a specific scientific quantitative measure. Traditional ROI is portrayed as a rational  measure of objectively assessed inputs leading to objective outputs [171]. This suggests that the scientific language of healthcare stakeholders for ROI is currently underdeveloped. It reflects general challenges of legitimising and aligning qualitative benefits with specific scientific measures that are seen as valid and trusted [48].

The QI literature discussed the use of ROI in several ways, including to create fiscal awareness. Such a use for ROI was noted by Botchkarev & Andru [29] in their analysis of ROI definitions. Their typology included the use of ROI as a persuasive device to gain credibility for a desired programme [9]. Healthcare leaders need credible recourse or language to articulate large-scale QI benefits [85, 170]. If we accept that reality is socially constructed, then we can view various logics as both coercive and emancipating [41]. That is, although political and market logics may constrain freedoms of local expressions, the mere tendency for humans to create their own meanings has potential to liberate from such constraints. Logics ‘in situ’ provide symbolic systems and vocabularies for expression. Hence, the prevailing logic both shapes and is shaped by contexts. Scientifically developing concepts for healthcare is essential to support this.

Establishing ways of expressing QI-ROI from healthcare programmes is crucial to avoid missing opportunities for essential healthcare improvements [30]. Additionally, insisting on inflexible use of a certain (ROI) policy may lead to data manipulation in bids to increase credibility [172, 173]. The view of ROI as both monetary and non-monetary benefits reflects the multi-stakeholder healthcare context. The lean towards non-monetary benefits is influenced by persistent healthcare and societal logics [11, 23, 47]. These logics emphasise relief of suffering and ethical principles such as beneficence (benefiting others) and non-maleficence (do no harm) [174]. It is therefore important to differentiate ROI as concept and as a metric. A concept is more than a metric, it encapsulates mental abstractions about how it is perceived by those using it and influences the decisions that then may follow [175].

## Strengths and limitations

This review has a few strengths and limitations. Concept analysis, concept development, and conceptual framework development are traditionally separate steps [55], unlike in this review. It is however accepted that these processes are intertwined [55]. However, we based our analysis on intensive background reading as well as a large review of different QI literature. This enabled us to gain some understanding of the current “state of the science” [55] surrounding the ROI concept as used in healthcare QI. We then followed a well-recognised Hupcey and Penrod [55], and Jabareen [53] development process to start to develop the concept QI-ROI in healthcare.

Secondly, productivity and efficiency proved to be crucial parts of the QI-ROI concept. These concepts were not included as search terms, however the large amount of literature retrieved means that it is unlikely that this made a significant difference in the review. Alternatively, it could be argued that our inclusion of specific ROI-like concepts in our search terms constitutes sampling bias. However, this strategy helped identify relevant literature for a more in-depth review. Thirdly, a significant amount of the literature reviewed was non-empirical in nature. Although this literature lacks a scientific focus, it was nonetheless very insightful in understanding the nature of the QI-ROI concept. Fourthly, some of the literature is quite dated, however newer literature suggest continuance of some trends and issues in QI-ROI and business case development. Lastly, subjectivity in the synthesis and analysis cannot be ruled out. As Parkinson et al. [176] put it “…findings are a consequence of intersubjective meaning-making through imagination, interpretation, and conceptual input…” (p15).

## Implications for research and practice

Implementation and Improvement Sciences are faced with the challenge of developing the ROI concept that is theoretically sound, and scientifically valid. This means a QI-ROI framework must clearly isolate constructs that can and should be included in an evaluation tool. The development of the QI-ROI concept and its conceptual framework must also ensure it is fit for purpose by incorporating both monetary and non-monetary benefits. This means finding more innovative and accessible ways for evaluating the QI-ROI aspects that are hard to measure and or monetise. Developing the QI-ROI concept in this way will enable the field to progress and take ownership in QI fiscal matters, and leaders to justify investments. This is crucial as justification for investment is unavoidable and necessary in the current economic climate.

The review indicated that the use of reporting tools is having a positive effect on the quality of QI studies. However, there remains room for improvement. QI researchers have a responsibility to show more transparency on ethical aspects of their studies. QI studies may not require ethical permissions, and if so, it must be stated as such. Current QI reporting tools allow for this [78, 148]. QI studies must also be clear about their statistical analysis methods use. Another area of improvement is the integration of qualitative and quantitative data in their analysis. This is important in strengthening research findings [177]. Further, reporting of study limitations was limited in the reviewed literature. The knowledge of QI implementation or research challenges can help arm other researchers and field practitioners in their QI initiatives. This is crucial for developing a stronger evidence-base as we develop the QI-ROI concept.

## Conclusion

Return-on-investment is an important tool with great potential to communicate QI benefits not covered by CEA and CBA. However, in its traditional form, ROI does not take advantage of this potential use. Ignoring the paradoxes contained within the traditional ROI use in healthcare may continue to keep ROI in the fringes of QI evaluation or cause conflict amongst stakeholders if enforced. Therefore, continued application of ROI must be based on its conceptualisation within healthcare QI and must be grounded on scientific inquiry that considers relevance to practice and policy. If QI-ROI is developed in this way, its legitimacy within healthcare stakeholders may be established and increased. In this review, we have begun to unpack what ROI is and means for healthcare stakeholders in QI at the organisational level. We hope to continue to develop this framework into a practical tool that is meaningful to its users: the QI teams and healthcare leaders, and QI investors.

## Supplementary Information

**Additional file 1: Supplementary Table 1**. Example search strategy. **Supplemental Table 2.** Data extraction tool. **Supplementary Table 3.** Included studies. **Supplementary Table 4.** Summary of Quality assessment.

## Data Availability

The datasets used and/or analysed during the current study are available from the corresponding author on reasonable request. Some data has been included in this article as its supplementary information files.

## Abbreviations

QI: Quality Improvement
ROI: Return on Investment
SROI: Social Return on Investment
QI-ROI: Return on Investment from healthcare quality improvement
CEA: Cost Effectiveness Analysis
CUA: Cost Utility Analysis
CBA: Cost Benefit Analysis
