# Correction: The development of the concept of return-on-investment from large-scale quality improvement programmes in healthcare: an integrative systematic literature review

**DOI:** 10.1186/s12913-022-08978-0

**Published:** 2022-12-22

**Authors:** S’thembile Thusini, Maria Milenova, Noushig Nahabedian, Barbara Grey, Tayana Soukup, Kia-Chong Chua, Claire Henderson

**Affiliations:** 1 King’s College London, London, UK; 2 South London and Maudsley NHS Foundation Trust, London, UK

**Correction: BMC Health Serv Res 22, 1492 (2022)**

**https://doi.org/10.1186/s12913-022-08832-3**

Following publication of the original article [1], the authors reported an error in the ‘Funding’ section.

The statement in the ‘Funding’ section originally read: No funding has been received for this study. The study is part of a PhD project by S’thembile Thusini, and the study is supported by UKRI. TS research is supported by the Welcome Trust (219425/Z/19/Z) and Diabetes UK (19/0006055). This work is supported by the Economic and Social Research Council, grant number ES/P000703/1.

The statement in the ‘Funding’ section should read: This work is supported by the Economic and Social Research Council, grant number ES/P000703/1. TS is supported by the Welcome Trust (219425/Z/19/Z) and Diabetes UK (19/0006055).

In addition, the authors reported typesetting mistake in the author list.

The incorrect author name is: S.’thembile Thusini

The correct author name is: S’thembile Thusini

The original article [1] has been updated.

## References

[1] Thusini (2022). The development of the concept of return-on-investment from large-scale quality improvement programmes in healthcare: an integrative systematic literature review. BMC Health Serv Res.

